# Open questions on the photophysics of ultrafast singlet fission

**DOI:** 10.1038/s42004-021-00527-w

**Published:** 2021-06-10

**Authors:** Justin C. Johnson

**Affiliations:** National Renewable Energy Laboratory, Golden, CO USA

**Keywords:** Excited states, Solar cells

## Abstract

Ultrafast singlet fission has the potential to facilitate highly efficient photovoltaics through the multiplication of excitons in organic molecular architectures. Here, we consider the interplay of molecular structure and intermolecular coupling toward enabling ultrafast singlet fission and discuss open questions in the field.

Singlet fission is a photophysical process by which a photoexcited singlet state generates two triplet excited states in proximal chromophores^1^. Early observations of this process date back more than half a century^2^ but until recently singlet fission seemed fated to anachronism. Renewed interest in the past decade is largely due to the recognition that singlet fission can potentially enhance solar energy conversion efficiency^3^.

Recent progress has been particularly strong toward the elucidation of the key intermediate in the scheme, the triplet pair (TT). Ultrafast and multidimensional laser spectroscopy, sophisticated organic synthesis, and powerful electronic structure calculations have combined to produce a detailed picture of triplet pair formation. Time-resolved electron paramagnetic resonance (EPR) spectroscopy has further carried the investigation of the triplet pair, from theoretical construct to experimental validation in the past half-decade. However, completion of the singlet-fission process requires that triplet pairs become independent species (i.e., two quasi-particles are born from one parent). Determining the rules governing this last step are leading to an unexpected richness of the photophysical tableau of coupled organic chromophores^4^.

Figure 1 shows the singlet-fission process in schematic form for the case of a periodic crystal (i.e., tetracene^5^). Molecules absorb light and produce a singlet excited state shared by several neighbors, in which some charge-transfer (CT) character may reside. Subsequently, two localized excitations both in the triplet state (TT) are born, which enables the S_1_ → TT conversion in some cases as fast as 100 fs. Lastly, the two triplets transfer among the molecules in the ensemble incoherently to become independent excitons (T + T). The details of each step, as they depend on molecular structure and intermolecular coupling, have begun to slowly emerge.

**Fig. 1:**
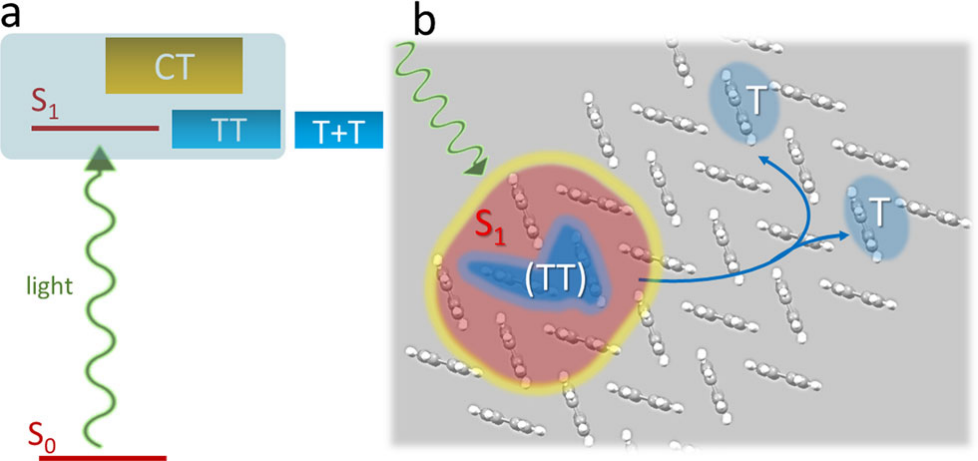
Schematic of the singlet-fission process. **a** Energy level arrangement of ground and excited singlet states (S_0_ and S_1_), triplet pair (TT), the independent triplets (T + T) and charge-transfer (CT) states. Teal shade indicates the possibility of state mixing. **b** Example of spatial arrangement and interconversion of species involved in ultrafast singlet fission, including initial delocalization of S_1_.

Singlet fission can proceed efficiently even on a picosecond or nanosecond timescale^6^. However, it is often the case that in a device other fast competing processes, such as energy and charge-transfer at molecule/semiconductor interfaces, occur from singlet states. This degrades the yield of triplets to lower than 200%, limiting singlet fission usefulness in practical situations^7^. All the same, the limits of ultrafast singlet-fission raise intriguing fundamental questions, because they may encroach on strong coupling and coherent regimes in molecular aggregates. Currently, the primary questions remaining to understand ultrafast singlet fission are related to how to reconcile classic mechanisms in the molecular photophysics of more familiar excited state processes (e.g., charge and energy transfer), with the uniqueness of singlet fission, namely the multiexcitonic nature of the singlet-fission products. Further, ostensibly extrinsic factors, such as the accompanying nuclear motions, have emerged as potentially crucial elements. Below we categorize the vanguard research in terms of the pressing questions that continue to drive researchers to investigate the singlet-fission process.

## What are the key factors that drive ultrafast singlet fission?

Under most circumstances, electronic state coupling between chromophores is a critical parameter that dictates the S_1_ → TT rate, via the Fermi’s Golden Rule^1^. In rare cases, this coupling is especially strong and the rate becomes coupling independent—the adiabatic regime^8^. An active area of singlet-fission research, both computational and experimental, is modulating interchromophore coupling through covalent dimer design and crystal engineering to test the bounds of nonadiabatic vs. adiabatic regimes and to engender both fast TT formation time and efficient TT dissociation. Although it is desirable to be in the strong coupling regime to quickly produce TT, completion of singlet fission to form T + T could still be hindered because a lowering of the S_1_ energy is often accompanied by strong coupling, resulting in a thermodynamic barrier^9^. Further, deleterious pathways from TT back to the singlet excited or ground state can be facilitated^10^.

The involvement of charge-transfer (CT) character in the photoexcited singlet is another major factor that has been implicated in ultrafast singlet fission, as it can lead to a facile route to triplet pairs through a so-called “mediated” mechanism^1^. The energy of the CT state can be controlled through molecular structure^11^, crystal engineering, and the solvent environment^12^. In a near-resonant situation, S_1_, CT, and TT states may all be accessible on sub-ps timescales, or mixed into the character of the initially photoexcited state. Ultrafast spectroscopy methods can often selectively probe the various species and resolve how they participate in the ultrafast singlet-fission mechanism.

The process of transforming between TT and the pair of independent triplets is often the rate determining step, and can be controlled through modifying the free-energy landscape between reactant and product. The larger the overall energy offset, or exothermicity, the faster the process tends to be, up to a limiting value^13^. Discovering and designing molecules with an appropriate exothermicity is a topic that has received considerable attention^14^. However, determining the rate dependence on exothermicity is more complex than measuring the enthalpy change of the overall transformation because the scheme can be arrested at the intermediate bound TT pair unless appropriate avenues for its separation are provided. Here, the factors involved are both enthalpic and entropic, and it is largely the pathways available toward triplet products isolated spatially that determine the speed of free triplet formation. Most polyacene derivatives with five or more benzene rings have ample enthalpic contributions (>0.1 eV) to undergo fast TT formation with even weak coupling, but the second step of singlet fission is only realized in chromophore architectures where triplets can separate efficiently^15^. Schemes for introducing many product microstates (i.e., delocalization in a crystal, or conformational states in a covalent system^16^) are now routinely applied to render TT formation ultrafast^17^ despite limited exothermicity and to assure efficient T + T production, sometimes on timescales of only a few ps.

## How do the spin states of the triplet pair influence free triplet formation?

The most commonly accepted model of the triplet pair has a manifold of nine spin sublevels into which population may flow after the first step of singlet fission^1^. Initially, the singlet-spin state ^1^(TT) forms due to the principle of conservation of spin angular momentum during the S_1_ → TT transformation. However, the population can evolve toward other spin states, including the intermediate spin ^3^(TT) and the highest spin quintet ^5^(TT). Which states ameliorate or strengthen the binding of the triplets to each other is largely dictated by the proximity and relative disposition of the pair of chromophores involved^18^. Time-resolved EPR spectroscopy has allowed researchers to investigate which of the various spin states the triplet pair can access and how the population evolves toward independent triplets. For example, ^3^(TT) may instigate an internal conversion process to a single triplet^19^, effectively annihilating one of the triplets of the pair and undermining the process. No such scheme exists for ^5^(TT), and thus in some cases it leads to unique long-lived EPR signatures and eventually independent triplets. Note, however that the time-resolution of EPR is limited to the nanosecond regime; therefore, these measurements are not a direct probe of ultrafast singlet fission^20,21^. Theoretical understanding of this process can currently offer only some qualitative guidance, because it requires various approximations, including limiting cases of strong or weak exchange coupling and high molecular symmetry. Progress in this area will require concurrent development of detailed models alongside incisive optical and magnetic resonance probes capable of tracking population dynamics on appropriate timescales in well-defined intermolecular juxtapositions^22^.

## What molecular motions accompany or instigate singlet fission?

**Fig. 2:**
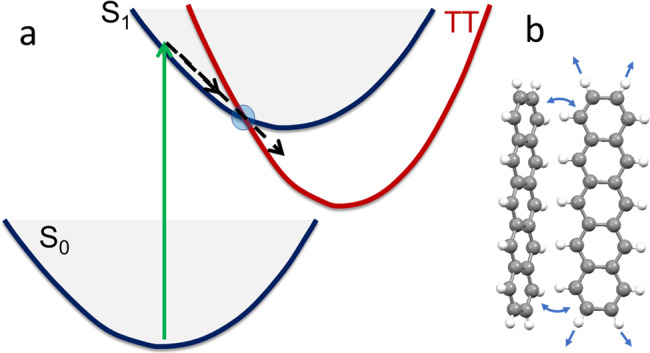
The role of geometrical rearrangements in singlet fission. **a** After photoexcitation (green) from S_0_, molecular motions (dashed) on the S_1_ potential energy surface propel the geometry toward the curve crossing with TT. **b** Both intramolecular (straight arrows) or intermolecular (curved arrows) motions can facilitate ultrafast conversion to the triplet pair.

The nuclear motions that accompany the transformation from S_1_ to TT and then to T + T are other limiting factors on the singlet-fission rate. In some cases, modes are predicted to break certain symmetries that make TT formation allowed^23^. In other scenarios, distance or conjugation modulated by intermolecular motions is suggested to improve the electronic coupling, creating a “gate” geometry at which singlet fission is ultrafast^24,25^. Intramolecular or intermolecular motions can also instigate fast singlet fission if there exists an intersection between S_1_ and TT potential energy surfaces (Fig. 2), through which population can funnel on timescales of ps or even fs^26^. The involvement of such vibronic modes in the electronic transformation are implicated in a variety of systems^27^, possibly circumventing the aforementioned need for strong electronic coupling in the equilibrium geometries of the dimer or crystal. If conditions are right the accompanying modes can be transmitted through the intersection and detected in the product electronic states through impulsive excitation and observation of vibrational coherences in pump-probe experiments^28,29^. The field has predominantly been exploring and discovering the influence of molecular motions, and rationally creating systems poised to take advantage of these effects will be a considerable but potentially impactful challenge for future investigations.

## Outlook

Although realizing the practical advantages of employing singlet fission in a solar photoconversion system possesses significant challenges, the foundational principles remain under intense investigation by a variety of scientists because of the rich diversity of effects encompassed. Precisely defined intermolecular geometries and experimental conditions will help to improve opportunities to assess comparative singlet-fission models that are currently under intense debate. In particular, more comprehensive models may need to be developed to understand singlet fission in the context of concurrent or competing processes that occur naturally in the bulk or at materials interfaces. Additional opportunities for triplet pair utility, such as in quantum information, will likely emerge as our understanding of dynamics becomes more complete.
